# Chronological and Spatial Distribution of Skeletal Muscle Fat Replacement in FHL1‐Related Myopathies

**DOI:** 10.1002/acn3.70258

**Published:** 2025-11-18

**Authors:** Rui Shimazaki, Satoru Noguchi, Hotake Takizawa, Yasushi Oya, Yuji Takahashi, Hirofumi Komaki, Hajime Arahata, Shinichiro Hayashi, Ichizo Nishino

**Affiliations:** ^1^ Department of Neuromuscular Research National Institute of Neuroscience, National Center of Neurology and Psychiatry Tokyo Japan; ^2^ Department of Genome Medicine Development Medical Genome Center, National Center of Neurology and Psychiatry Tokyo Japan; ^3^ Department of Neurology National Center Hospital, National Center of Neurology and Psychiatry Tokyo Japan; ^4^ Department of Child Neurology National Center Hospital, National Center of Neurology and Psychiatry Tokyo Japan; ^5^ Translational Medical Center, National Center of Neurology and Psychiatry Tokyo Japan; ^6^ Department of Neurology Neuro‐Muscular Center, NHO Omuta Hospital Omuta Japan

**Keywords:** Emery‐Dreifuss muscular dystrophy, FHL1‐related myopathies, modified Mercuri score, reducing body myopathy, whole‐body muscle imaging

## Abstract

**Objectives:**

Variants in the *FHL1* gene cause FHL1‐related myopathies (FHL1‐RMs), a group of neuromuscular disorders with diverse clinical presentations. This study aimed to comprehensively characterize the spatial and temporal patterns of skeletal muscle fat replacement throughout the whole body in FHL1‐RMs, to examine disease progression over time, and to evaluate the relationship between imaging findings and clinical symptoms.

**Methods:**

We retrospectively analyzed 21 whole‐body imaging studies from 10 patients with genetically confirmed FHL1‐RMs. Fatty replacement was scored in 47 muscles using the modified Mercuri score (mMS). Longitudinal data were used to stratify patients into slow, moderate, and rapid progression groups. *K*‐means clustering was applied to classify muscles based on their chronological patterns of fatty infiltration. Hierarchical clustering and violin plots were used to explore inter‐muscle and inter‐patient variations.

**Results:**

Despite notable variability in the rate of disease progression, a consistent pattern of muscle involvement was observed across patients. Muscles were classified into three progression clusters: early‐onset and early attainment of the maximal mMS (e.g., paraspinal and posterior thigh muscles), steadily progressive (e.g., trunk and lower leg muscles), and late‐onset with slow changes (e.g., shoulder and anterior thigh muscles). These patterns paralleled the clinical symptom progression. In early‐stage patients, STIR imaging revealed muscle signal abnormalities preceding fat replacement detectable on T1‐weighted images.

**Interpretation:**

The rate of fat replacement in FHL1‐RMs varies individually, but spatial patterns are conserved and reflect clinical evolution. Serial imaging is a valuable tool to monitor disease progression and may serve as a sensitive biomarker in clinical trials.

## 1 Introduction

The four and a half LIM domains 1 (*FHL1*) gene encodes a protein containing a zinc finger domain and a half LIM domain (derived from Lin‐11, Isl‐1, and Mec‐3) at its N‐terminus, followed by four complete LIM domains [1]. FHL1 protein together with myosin‐binding protein C, plays a crucial role in sarcomere assembly [2]. Variants in the *FHL1* gene cause FHL1‐related myopathies (FHL1‐RMs), and FHL1‐RMs include a variety of neuromuscular disorders. Missense variants affecting cysteine or histidine residues involved in the formation of zinc finger structures within the LIM domains lead to abnormal protein aggregation. These aggregates sequester wild‐type *FHL1*, exerting a dominant‐negative effect, and result in reducing body myopathy (RBM) [1, 3]. Additionally, variants in *FHL1* have been associated with diverse phenotypes, including X‐linked myopathy with postural muscle atrophy, scapuloperoneal myopathy, rigid spine syndrome (RSS), and Emery‐Dreifuss muscular dystrophy (EDMD) [4, 5, 6, 7]. Notably, EDMD is known to result from loss‐of‐function variants in *FHL1* [8].

Skeletal muscle imaging plays a critical role in the diagnosis and investigation of neuromuscular diseases [9]. In hereditary myopathies, each disease typically demonstrates a characteristic pattern of fatty replacement, which not only aids in differential diagnosis but can also serve as an inclusion criterion or outcome measure in clinical trials [10]. The pattern of fatty replacement in leg muscle of patients with FHL‐RMs has been previously described. Imaging findings revealed a “geographical pattern” with neighboring areas within the same muscle showing strikingly different degrees of fat replacement; areas showing low attenuation on CT and high signal intensity on T1‐weighted MRI were adjacent to regions that appeared normal. This pattern has also been observed in VCP myopathy [11].

This study aims to comprehensively characterize the whole‐body distribution of muscle fatty replacement in FHL1‐RMs, to investigate the chronological progression of fatty replacement, and analyze its association with clinical findings. Through these analyses, we seek to evaluate the potential utility of imaging data as a clinical biomarker in clinical practice.

## 2 Methods

### 2.1 Patients

We selected 10 patients with pathogenic variants in the *FHL1* gene and available whole‐body skeletal muscle imaging data from the NCNP repository, collected for diagnostic purposes between January 2002 and October 2024. Among them, six patients underwent multiple imaging studies at different time points, resulting in a total of 21 imaging datasets being evaluated. Clinical information for these patients was also collected.

### 2.2 Data Collection

The collected clinical information included sex, age at onset, disease duration at the time of imaging, muscle biopsy site, clinical symptoms, joint contractures, winged scapula, spinal rigidity, cardiac symptoms, reducing bodies, and genetic variants.

### 2.3 Disease Classification

Patients were diagnosed based on clinical findings, pathological findings, and genetic information. Specifically, patients presenting with reducing bodies or those carrying previously reported variants associated with RBM were classified as RBM. Patients exhibiting spinal rigidity were classified as RSS, and patients with truncating variants, in addition to presenting with myopathy and either cardiac involvement or joint contractures, were classified as EDMD. Muscle pathology was performed based on biopsy specimens obtained from the biceps brachii (BB), quadriceps femoris (QF), and deltoid (Del) muscles.

### 2.4 Muscle Imaging Analysis

We obtained 21 whole‐body imaging studies from 10 individuals. The details of the imaging modalities for each case are provided in the Table S1. In accordance with our previous study, we confirmed the interchangeability between CT scan and MRI scan [12]. Axial images from CT and/or T1‐weighted MRI were used to assess the degree of fatty replacement in each muscle at the level of maximal cross‐sectional area, based on the modified Mercuri score (mMS, range 0–4) as previously described [12]. A total of 47 muscles were evaluated, as listed below:

Neck: Cervical paraspinal muscles (CPs), sternocleidomastoid (Scm), levator scapulae (LS), and splenius capitis (SC).

Arm and shoulder: Pectoralis major (PM), wrist flexors (WF), wrist extensors (WE), triceps brachii (TB), BB, Del, infraspinatus (Infs), subscapularis (Subs), thoracic paraspinal muscles (TPs), rhomboids (Rh), and trapezius (Tpz). Due to insufficient resolution, individual forearm muscles were not analyzed separately and were evaluated as WF and WE.

Trunk: Latissimus dorsi (LD), serratus anterior (SA), multifidus (Mf), longissimus (Lo), iliocostalis lumborum (IcL), external abdominal oblique (EAO), internal abdominal oblique (IAO), and rectus abdominis (RA).

Pelvis: Iliopsoas (IP), gluteus maximus (GMx), gluteus medius (GMd), gluteus minimus (GMn).

Thigh: Rectus femoris (RF), sartorius (Sar), gracilis (Gr), vastus lateralis (VL), vastus medialis (VM), vastus intermedius (VI), adductor longus (AL), adductor magnus (AM), semimembranosus (Sm), semitendinosus (St), short head of the biceps femoris (BFS), and long head of the biceps femoris (BFL).

Calf: Tibialis anterior (TA), tibialis posterior (TP), extensor digitorum longus (EDL), fibularis longus (FL), fibularis brevis (FB), soleus (Sol), medial head of the gastrocnemius (GcM), and lateral head of the gastrocnemius (GcL).

When there was a difference in laterality in mMS, the higher score was recorded. A laterality difference in fatty replacement was considered present when the mMS differed by two or more points between the left and right sides.

### 2.5 Data Analysis

For the patients who underwent imaging assessments at multiple time points, the relationship between total mMS and disease duration was analyzed and the patients were classified into three groups with different progression rates according to the annual increase in total mMS:

Slow progression group: 1–9 mMS increase/year.

Moderate progression group: 10–20 mMS increase/year.

Rapid progression group: > 20 mMS increase/year.

To classify the muscles which contribute mMS increment on chronological data, *K*‐means clustering was conducted based on the pattern of mMS changes. Among the muscles belonging to the same cluster, the average mMS at each imaging time point was calculated and plotted. Regression lines were drawn and analyzed to deduce the relationship between disease duration and mMS. *K*‐means clustering was performed using the R software (version 4.2.1; R Core Team, 2022). We also performed hierarchical clustering based on whole‐body mMS data. To describe the progressive changes of fatty replacement of each muscle, we applied violin plots using GraphPad Prism 10.2.0 (GraphPad Software, San Diego, CA) and compared them between groups.

### 2.6 Statistical Analysis

Association between continuous variables was assessed using regression analysis. Sum of mMS (dependent variable) and disease duration (independent variable), linear and logarithmic regression analysis; association between mean mMS per progression cluster (dependent variable) and disease duration (independent variable), linear and logarithmic regression analyses; comparison of the number of muscles with laterality of mMS between male and female patients, the Mann–Whitney U test; comparison of mMS between groups, one‐tailed Welch's *t*‐test. A *p*‐value of < 0.05 was considered statistically significant. Power and sample size calculations were not conducted due to small sample size.

### 2.7 Ethical Approval and Consent

This study was approved by the Ethics Committee of the NCNP (approval number: A2022‐045). Written informed consent was obtained from all participants for the use of their data in research.

## 3 Results

**FIGURE 1:**
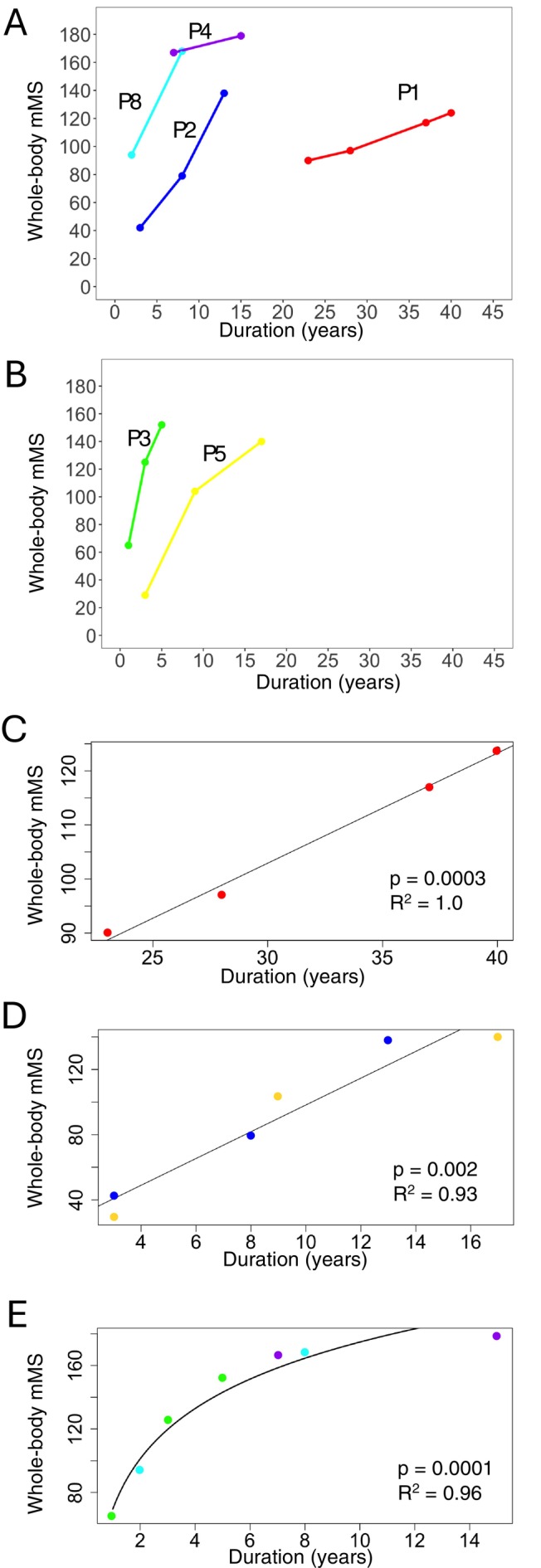
Relationship between imaging data and disease duration in patients with FHL1‐related myopathy. Scatter plot showing the relationship between total mMS and disease duration in patients who underwent multiple imaging examinations (*N* = 17). (A) Female patients. (B) Male patients. Patients are shown by color. (C–E) Correlation between total mMS and disease duration among patients classified according to progression rate. Strong correlations were observed in all groups with different slopes: (C) slow patient group, (D) moderate patient group, and (E) rapid patient group. The correspondence colors are as follows: Red = P1, blue = P2, green = P3, purple = P4, amber = P5, light blue = P8.

**FIGURE 2:**
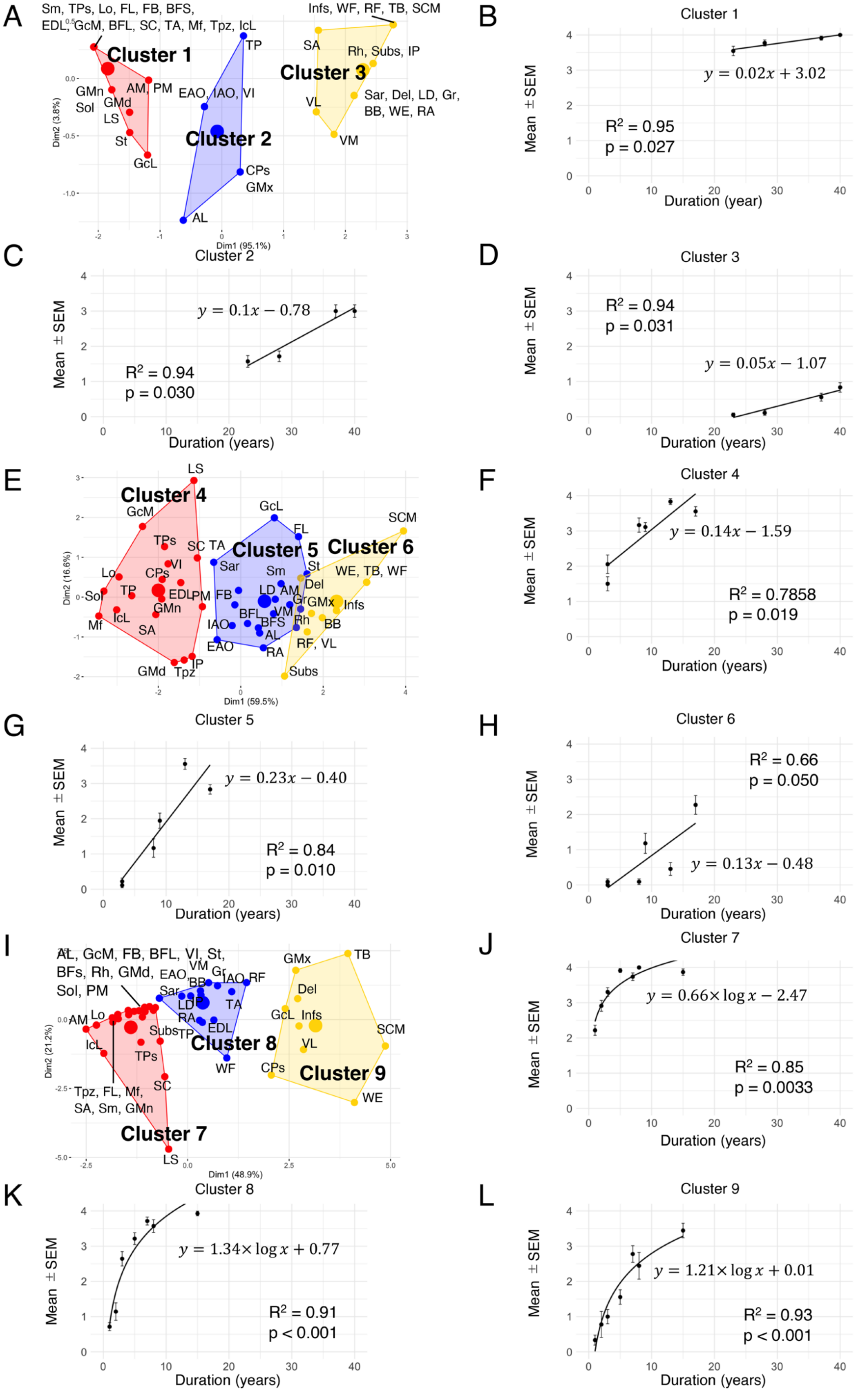
Chronological fatty replacement pattern in each muscle in FHL1‐RMs. Patients were divided into three groups according to disease progression (A) Slow patient group, (E) Moderate patient group, and (I) Rapid patient group, and muscles were classified into three clusters (Pattern 1: Red, Pattern 2: Blue, Pattern 3: Yellow) using *k*‐means based on longitudinal mMS changes. Slow patient: Defined as 1–9 mMS increase/year. Slow patient group consists of P1. Moderate patient: Defined as 10–20 mMS increase/year. Moderate patient group consists of P2 and P5. Rapid patient: Define as > 20 mMS increase/year. Rapid patient group consists of P3, P4 and P8. To describe the cluster‐specific progression patterns, the mean mMS values of all muscles involved in each cluster were plotted at the imaging time points, and regression lines were drawn to estimate a slope and *y*‐axis intercept. Panels (B, C, D) represent chronological changes of clusters 1, 2, and 3; panels (F, G, H) represent those of clusters 4, 5, and 6; panels (J, K, L) represent those of clusters 7, 8, and 9, respectively.

The characteristics of the patients were summarized in Table 1. Scatter plots of chronological imaging data were generated separately for male and female FHL1‐RMs patients, who represent RBM in muscle and underwent multiple imaging studies, by disease duration and the sum of mMS (Figure 1A,B). Although slopes and intercept values varied among individual patients, fat replacement progressed linearly in all patients. We classified them into three patient groups with different progression speeds based on their annual increase in the sum of mMS: slow progression group: P1; moderate progression group: P2 and P5; rapid progression group: P3, P4, and P8. Each group was confirmed to display a strong correlation between the sum of mMS and disease duration (Figure 1C–E). Next, we conducted *k*‐means clustering of the muscles based on mMS change patterns on duration in each group. We plotted the mean mMS changes on disease duration in each *k*‐means cluster. From regression lines, the progressive pattern of fat replacement in each colored cluster was similar across the groups (Figure 2). “Red” clusters showed higher *y*‐axis intercept and low slope, representing muscles affected in the early stages of the disease, which show the higher mMS at early stage and do not increase further as it progresses (Pattern 1: Clusters 1, 4, and 7). “Blue” clusters showed low *y*‐axis intercept and high slope, representing muscles that show substantial increases in mMS at late stage (Pattern 2: Clusters 2, 5, and 8), and “Yellow” clusters showed low *y*‐axis intercept and low slope, representing muscles with slow progression and minimal changes in mMS (Pattern 3: Clusters 3, 6, and 9). Based on colored clustering results in the grouping data, we classified the muscles into three groups by majority rule (Table S2). These results classified that the calf muscles, posterior thigh muscles, and paraspinal muscles belonged to “Red cluster”, trunk muscles to “Blue cluster”, and shoulder and arm muscles to “Yellow cluster.”

**TABLE 1:** List of patients with FHL1‐related myopathies.

Patient	Disease	Sex	Onset (y.o)	Biopsy site	Cardiac symptom	Contracture	Rigid spine	Winged scapula	Reducing body	Variant (FHL1:NM_001449.4)	Other	Image ID	Duration at imaging (year)
P1	RBM	F	32	L, BB	No	No	No	No	Yes	c.304_312delAAGGGGTGC p.(K102_C104delKGC), reported [13]	Mother of P2	P1‐1	23
												P1‐2	28
												P1‐3	37
												P1‐4	40
P2	RBM	F	23	N.A	No	No	No	No	N.D	c.304_312delAAGGGGTGC p.(K102_C104delKGC), reported [13]	Daughter of P1	P2‐1	3
												P2‐2	8
												P2‐3	13
P3	RBM	M	10	L, BB	IRBBB	Ankle, knee, elbow	No	No	Yes	c.310T>C p.(C104R), reported [13]	Son of P4	P3‐1	1
												P3‐2	3
												P3‐3	5
P4	RBM	F	29	L, BB	No	No	No	No	Yes	c.310T>C p.(C104R), reported [13]	Mother of P3	P4‐1	7
												P4‐2	15
P5	RSS	M	13	L, BB	No	No	Yes	Yes	Yes	c.451_459delGTGACTTGC p.(V151_C153delVTC), reported [14]		P5‐1	3
												P5‐2	9
												P5‐3	17
P6	RBM	M	1	L, RF	No	No	No	No	Yes	c.386G>A:p.(C129Y), reported [15]		P6	0
P7	RBM	M	31	R, RF	No	No	No	No	Yes	c.394T>C p.(C132R), reported [16]		P7	6
P8	RBM	F	16	L, BB	No	No	No	Yes	No	c.449G>A p.(C150Y), reported [13, 17]		P8‐1	2
												P8‐2	8
P9	EDMD	M	20	L, deltoid	No	Ankle	No	Yes	No	c.810delA p.(V271Cfs × 19), unreported		P9	33
P10	EDMD	M	41	L, QF	HCM	No	No	No	No	c.469_470del p.(K157Vfs × 36), reported [5]		P10	6

Abbreviations: EDMD, Emery‐Dreifuss muscular dystrophy; HCM, hypertrophic cardiomyopathy; IRBBB, incomplete right bundle branch block; N.A, not available; N.D, no data; QF, quadriceps femoris; RBM, reducing body myopathy; RSS, rigid spine syndrome; y, year; y.o, years old.

Next, we transversely overviewed all 21 imaging data we obtained including the patients with multiple‐time points images and those with single‐point image on a heat‐map. Fatty replacement (mMS ≥ 1) was observed in 20 out of 21 imaging studies (95.2%) in the TP, GcM, VI, IcL, Lo, and Mf. In the AM, GMn, SA, and CPs, fatty replacement was observed in 19 out of 21 studies (90.5%). As described in previous studies, muscles tended to exhibit a “geographical” pattern on imaging studies [11]. In contrast, fatty replacement was less frequently seen in the RF, TB, WE, and SCM, being present in 8, 5, 8, and 5 out of 21 studies (38.1%, 23.8%, 38.1%, and 22.8%), respectively. Regarding laterality differences in mMS, left–right differences were observed (Table S3). The median number of muscles with laterality differences was 1 in males and 7 in females, and this difference was statistically significant (Table S4) (Mann–Whitney U test, *p* = 0.00034).

**FIGURE 3:**
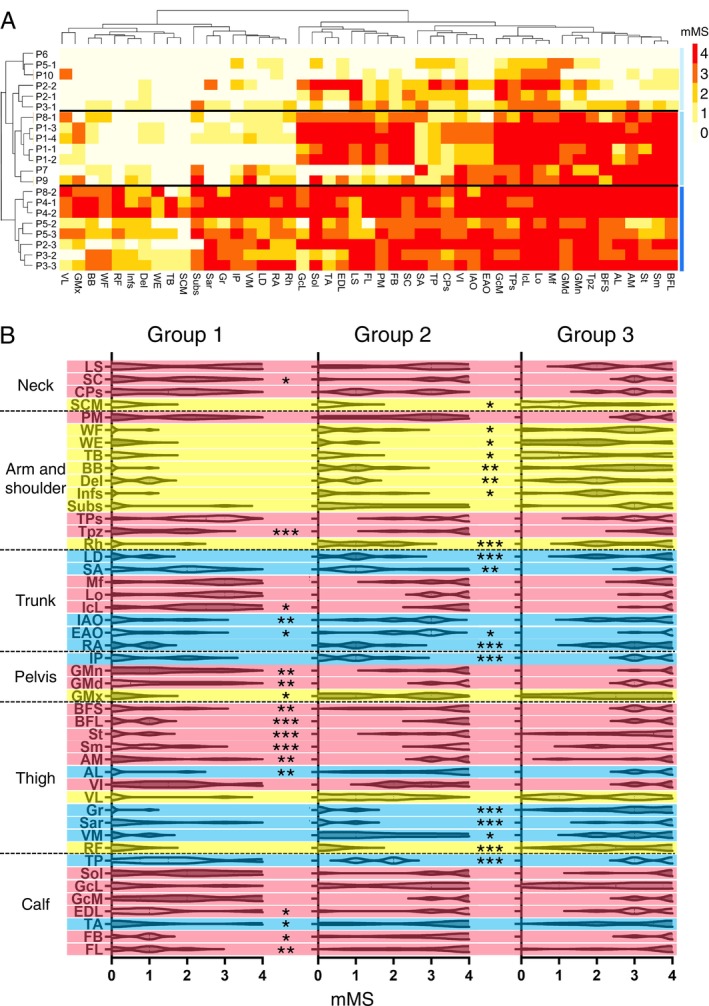
Hierarchical clustering of patients by whole‐body skeletal muscle imaging data and violin plots. (A) A heatmap of imaging data in whole‐body skeletal muscles. Muscles (denoted by capital alphabets in the bottom) are aligned horizontally, and images (denoted by the number on the left) are aligned vertically by hierarchical clustering. The mMS of the muscles was shown in color denoted in the right scale. From top to bottom, patients were classified into three groups (Groups 1–3) by sum of mMS. Light blue indicates low‐total mMS score group (Group 1). Medium blue indicates medium‐total mMS group (Group 2). Dark blue indicates high‐total mMS group (Group 3). (B) The distribution of mMS in each muscle in each group (Group 1–3) on violin plots. The data of mMS were compared between neighbor two groups using a *t*‐test. *p < 0.05, **p < 0.01, ***p < 0.001. The temporal progression patterns of muscle fat replacement shown in Figure 2 are represented in violin plots with overlaid colors. Red indicates Pattern 1, blue indicates Pattern 2, and yellow indicates Pattern 3.

**FIGURE 4:**
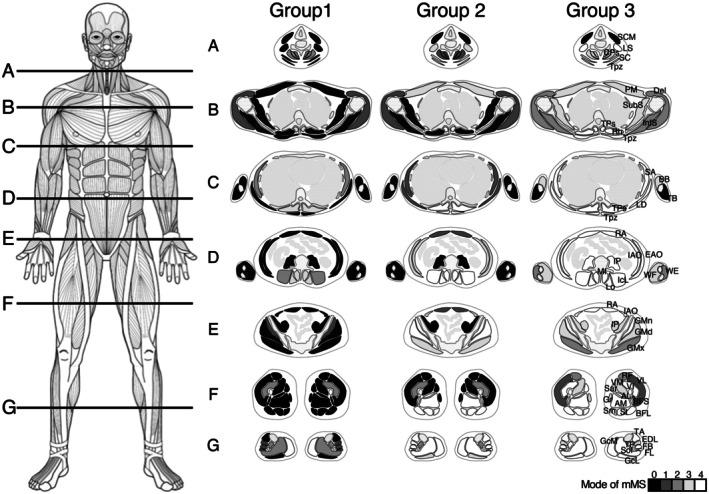
Illustration of fatty replacement patterns of whole‐body muscles at three disease stages. Graphical representation of mode of the mMS among corresponding patients by cross‐sectional views from the neck to calf. (A) neck level, (B) shoulder level, (C) chest level, (D) abdominal level, (E) pelvis level, (F) thigh level, (G) calf level. mMS is shown in gray scale as denoted in the right scale.

By hierarchical clustering, the image data were classified into three branches on the left dendrogram (Groups 1–3, with low to high total mMS) (Figure 3A). Each group, Groups 1–3, showed its specific muscle involvement pattern and contained images at earlier stages (P2‐1, P2‐2, P3‐1, and P5‐1), those at mid stages (P1‐1, P1‐2, P1‐3, P1‐4, and P8‐1) and those at later stages (P2‐3, P3‐2, P3‐3, P4‐1, P4‐2, P5‐2, P5‐3, and P8‐2), respectively from different progression rate groups in Figure 1, showing that the muscle involvement patterns were quite similar across the patients and were determined by disease stages, but not related to progression rates. P9 and P10 have a truncating variant and represent EDMD phenotypes. P9 exhibited joint contractures, while P10 presented with hypertrophic cardiomyopathy, a condition that has been reported to be frequently associated with FHL1‐related EDMD [5]. They were classified into the same clusters as the patients who have cysteine substitution and RBM pathology. Furthermore, the muscle clustering classified them into three branches as shown at the top. The left branch contains mainly the muscles belonging to the Yellow cluster, the center contains those in the Blue cluster and the right contains those in the Red cluster. Again, the longitudinal chronological changing pattern of each muscle was maintained across patients. We tried to identify the spatial and temporal patterns in the fat replacement across clustered groups using a violin plot. Additionally, we colored each muscle with those corresponding clusters which show the different kinetics in fat replacement as illustrated in Figure 2 (Figure 3B). Because CPs, SA, IP, BFS, GcL muscles were classified into three different colored clusters in each progression patient group, the colored clusters were determined based on the progression patterns in the violin plots. The coloring classification strongly matched with the progressive patterns of fat replacement in each muscle on violin plots, showing the presence of muscle‐specific fat replacement kinetics. Interestingly, almost all neck muscles, paraspinal muscles, posterior leg muscles belonged to the Red cluster; arm and shoulder muscles and anterior thigh muscles belonged to the Yellow cluster; and trunk muscles, medial calf muscles and anterior calf muscles belonged to the Blue cluster, each of which represented significant progression in fat replacement at earlier stages, consistently and at later stages, respectively. An illustration shows the specific pattern of fat replacement at progression stages (Figure 4).

To investigate the association between disease progression patterns based on imaging findings and clinical symptoms, we summarized the order of appearance of the symptoms in each patient in Table 2. Initial symptoms appeared in the legs or trunk, followed later by symptoms in the arms in almost all patients. This order appeared to be consistent with the pattern of muscle fatty replacement. Additionally, joint contractures, spinal rigidity, and winged scapula were observed in one to three out of 10 individuals, which would be related to fat replacement in the paraspinal spinal muscles and Tpz.

**TABLE 2:** Clinical data in each patient.

Patient	First symptom/second/third
P1	Leg weakness (32 y.o)/arm weakness (42 y.o)
P2	Leg weakness and spinal rigidity (23 y.o)
P3	Leg weakness (10 y.o)
P4	Leg weakness (29 y.o)/arm weakness (30 y.o)
P5	Leg weakness (13 y.o)/spinal rigidity (15 y.o)
P6	Leg and trunk weakness (1 y 5 m)/arm weakness (1 y 6 m)
P7	Trunk weakness (31 y.o)/Leg weakness (34 y.o)/arm weakness (37 y.o)
P8	Leg weakness (16 y.o)/arm weakness (16 y.o)
P9	Leg weakness (20 y.o)/arm weakness (48 y.o)
P10	Leg weakness (41 y.o)

Abbreviations: m, months; y.o, years old.

**FIGURE 5:**
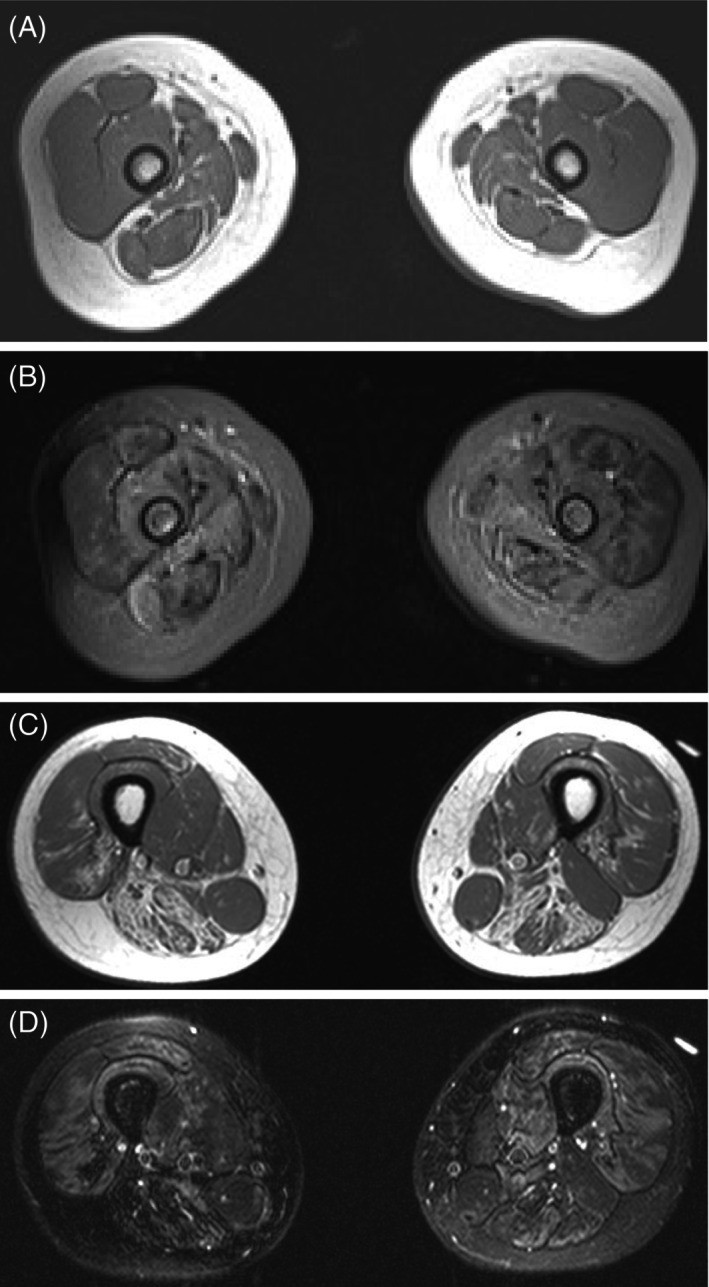
Representative T1‐weighted and STIR muscle MRI showing fatty infiltration and edema. Axial thigh MRI images of patients P6 and P8. (A) T1‐weighted image of P6. (B) STIR at same level as (A). (C) T1‐weighted image of P8. (D) STIR at same level as (C).

Because P6 and P8 were still at an earlier stage in fat replacement, enough information for the evaluation on CT or T1‐weighted MRI was not obtained. Therefore, thigh MRI Short Tau Inversion Recovery (STIR) images were acquired. P6 exhibited hyperintensity in the RF, VL, VM, VI, Sar, Sm, St, BFL, BFS, and AM muscles. P8 showed hyperintensity predominantly in the VL, VM, and VI muscles. Interestingly, the intense signals were obtained, even though those muscles exhibited minimal fat replacement (Figure 5).

## 4 Discussion

A key strength of this study was the imaging assessments for individual patients at longitudinal multiple time points. Longitudinal analysis of muscle imaging and their clustering technologies allowed us to identify the temporal and spatial patterns of muscle fat replacement in FHL1‐RMs. A notable characteristic of FHL1‐RMs is the substantial variability in both age of onset and disease progression kinetics [18]. In our cohort, the age of onset ranged widely from 1 to 41 years, and even when considering only patients with missense and in‐frame variants, onset varied between 1 and 31 years. Interestingly, despite this variability in progression rates (slopes), the total mMS demonstrated linear progression along aging. Chronological imaging analysis revealed predominant involvement of muscles in the posterior thigh, lower leg, and paraspinal region during the early stage, progression to muscles of the arms and shoulders in the late stage, and continuous fat replacement progression in trunk muscles throughout all stages.

What makes the progression rate of fat replacement? Sex may be one of the factors that influence the disease progression rate. In this study, P3 and P4, a mother–her son, showed nearly a 20‐year difference in the age of onset. Previous studies have also reported milder disease phenotypes in females [13, 14, 19]. The milder phenotypes in females are thought to be associated with X‐chromosome inactivation where the *FHL1* gene is present. It could also explain the significant asymmetry in fat replacement observed in female patients [11]. However, we found that female patients, P2 and P8 similarly showed the elevation of total mMS to male P3 and P5. These facts suggest the presence of modifying factors other than genetic variation that determine progression rates.

The patients with EDMD were classified into the same branches as those with RBM in hierarchical clustering. RBM and EDMD have been considered to be different diseases and are caused by different variants: cysteine substitution in the second LIM domain and protein‐truncating variants, respectively [12], which have different pathogenicity. In fact, the muscle pathology in these patients was different. Interestingly, however, the spatial patterns of fat replacement observed in imaging were similar in both patients. Longitudinal observation and comparison of clinical symptoms between patients with different variants are required.

Previous reports have described FHL1‐RMs as typically presenting with symptoms such as gait disturbances related to leg weakness and spinal rigidity [20]. The clinical course observed in our cohorts aligns with these reports, as the symptoms initially manifest in the legs and trunk and subsequently progress to the arm and shoulder. Our findings suggest that clinical symptoms and imaging changes may progress in parallel. Previous studies have also reported that imaging can detect muscle degeneration more sensitively than clinical symptoms [21]. Therefore, monitoring disease progression through imaging may enable the early and sensitive detection of disease stage and symptom progression. Cardiomyopathy was observed in one patient (P10) carrying a truncating variant. Cardiomyopathy related to *FHL1* variants has been reported previously in cases harboring truncating variants, suggesting that the variant type may be associated with cardiac involvement [22, 23, 24].

In the younger patients, areas without evident fat replacement exhibited high‐intensity signals on STIR imaging. It is consistent with the findings in previous studies [15, 25]. It is noteworthy that P6, who was only 1 year old, exhibited clinical symptoms, but fat replacement was undetectable on T1‐weighted MRI, while high‐intensity signals on STIR imaging were observed. This observation suggests that fat replacement may appear later than muscle weakness. Since STIR can monitor the affected muscles at an earlier stage, it might allow early diagnosis and therapeutic intervention to FHL1‐RMs.

Our study could potentially provide a valuable measurement tool for assessing therapeutic outcomes, such as those in future therapy trials. Specifically, by comparing the trajectory of fat replacement scores over time with/without an intervention, it may be possible to evaluate therapeutic efficacy. Furthermore, the imaging may serve as an objective measure of the muscle status of the attending patients for setting a useful inclusion criterion for clinical studies. Grouping patients appropriately based on their disease progression rates could further enhance the accuracy of therapeutic efficacy assessments. Therefore, performing imaging assessments at multiple time points with defined intervals in a single individual could be beneficial to determine the patients' fat replacement progression rates.

There are limitations in this study. (1) The available clinical information on the patients was limited. This made it challenging to establish the correlation between imaging findings and the various clinical symptoms in this disease. (2) As FHL1‐RMs are rare disorders, the number of cases with available imaging data was limited. In particular, EDMD cases are extremely rare, and thus only two cases were included in this study. Given the variability in the progression rate among the patients with this disease, a larger sample size using the same modality imaging data obtained at regular time intervals would have been ideal for analysis. The small sample size limits the study to an exploratory analysis. Furthermore, because multiple imaging data points from the same patient were treated as independent, some bias in the distribution of fat replacement may have occurred. (3) In some cases, only partial imaging data were available; therefore, the degree of fatty replacement may have been either overestimated or underestimated.

In the current study, we found that the chronological progression varied among individual patients, though a consistent spatial pattern of fat replacement was observed. This finding may be relevant for establishing inclusion criteria and assessing the efficacy of future therapy trials. These data could be used as surrogate outcome measures in upcoming clinical trials for FHL1‐RMs.

## Author Contributions

**Rui Shimazaki:** conceptualization, data curation, formal analysis, investigation, methodology, writing – original draft, validation, software, visualization. **Satoru Noguchi:** investigation, methodology, visualization, writing – review and editing, validation, supervision. **Hotake Takizawa:** collecting the data sets. **Yasushi Oya:** collecting the data sets. **Yuji Takahashi:** collecting the data sets. **Hirofumi Komaki:** collecting the data sets. **Hajime Arahata:** collecting the data sets. **Shinichiro Hayashi:** conceptualization, writing – review and editing, validation, supervision. **Ichizo Nishino:** conceptualization, writing – review and editing, project administration, validation, supervision.

## Disclosure

We confirm that we have read the Journal's position on issues involved in ethical publication and affirm that this report is consistent with those guidelines.

## Conflicts of Interest

The authors declare no conflicts of interest.

## Supporting information


**Table S1:** acn370258‐sup‐0001‐TableS1.docx.

**Table S2:** acn370258‐sup‐0002‐TableS2.xlsx.

**Table S3:** acn370258‐sup‐0003‐TableS3.docx.

**Table S4:** acn370258‐sup‐0004‐TableS4.xlsx.

## Data Availability

The data that support the findings of this study are available from the corresponding author upon reasonable request.
