# Supplementary material for: Chronological and Spatial Distribution of Skeletal Muscle Fat Replacement in FHL1‐Related Myopathies
Source: Ann Clin Transl Neurol. 2025 Nov 18;13(4):724–35. doi: 10.1002/acn3.70258 (PMC13071098; doi:10.1002/acn3.70258)
Supplement: Supplementary file 1 — Table S1: acn370258‐sup‐0001‐TableS1.docx. [file ACN3-13-724-s004.docx]

Supplementary Table 1 Imaging study protocol

| **Patient** | **modality** | **Anatomical region** | **Protocol** | **Thickness (mm)** | **Tube voltage (kVp)** |
| --- | --- | --- | --- | --- | --- |
| P1 | CT | whole-body (continuous) | helical | 5 | 120 |
| P2 | CT | whole-body (continuous) | helical | 5 | 120 |
|  | MRI | thigh, calf | T1 weighted imaging | 8 |  |
| P3 | CT | whole-body (segmental) | axial | 10 | 120 |
| P4 | CT | whole-body (segmental) | axial | 10 | 120 |
| P5 | CT | whole-body (segmental) | axial | 10 | 120 |
| P6 | CT | whole-body (segmental) | axial | 8 | 120 |
|  | MRI | calf, lumbar | T1 weighted imaging | Calf 7  Lumbar 4 |  |
| P7 | CT | whole-body (continuous) | helical | 5 | 135 |
| P8 | CT | whole-body (continuous) | helical | 5 | 120 |
|  | MRI | upper arm, thigh | T1 weighted imaging | Upper arm 6  Thigh 8 |  |
| P9 | CT | whole-body (continuous) | helical | 5 | Not available |
| P10 | CT | whole-body (continuous) | helical | 10 | 120 |
