# Supplementary material for: Chronological and Spatial Distribution of Skeletal Muscle Fat Replacement in FHL1‐Related Myopathies
Source: Ann Clin Transl Neurol. 2025 Nov 18;13(4):724–35. doi: 10.1002/acn3.70258 (PMC13071098; doi:10.1002/acn3.70258)
Supplement: Supplementary file 3 — Table S3: acn370258‐sup‐0003‐TableS3.docx. [file ACN3-13-724-s003.docx]

**Supplementary Table 3 List of muscles showing asymmetric fat infiltration**

| **Image ID** | **Muscles showing asymmetric fat infiltration** |
| --- | --- |
| P1-1 | GcL, Sol, VI, AM, BFL, IAO, EAO, PM, SC, LS |
| P1-2 | GcL, VI, AM, St, BFL, IAO, EAO, PM, SC, LS |
| P1-3 | GcL, VI, AL, BFL, GMx, IAO, EAO, PM, SC, LS |
| P1-4 | GcL, VL, VI, VM, AL, AM, GMx, IAO, EAO, PM, SC, LS |
| P2-1 | GcM, VI, LS |
| P2-2 | TP, GcL, Sol, VI, Sar, GMd, IAO, EAO, LS |
| P2-3 | GcL, VI, Sar, Gr, EAO, LS |
| P3-1 | FL, Sm |
| P3-2 | FL, FB, Sar |
| P3-3 | No asymmetric fat infiltration |
| P4-1 | RF, Sar, Gr, IP, Rh, Subs, TB |
| P4-2 | Rh, Subs |
| P5-1 | IP |
| P5-2 | FB, TA |
| P5-3 | No asymmetric fat infiltration |
| P6 | No asymmetric fat infiltration |
| P7 | GcM, VI, VM |
| P8-1 | TA, GcL, BB, PM |
| P8-2 | TP, Sol, LD, Subs, Infs |
| P9 | EDL |
| P10 | No asymmetric fat infiltration |
